# The Incidence of Radial Artery Occlusion in Critically Ill Patients after Cannulation with a Long Catheter

**DOI:** 10.3390/jcm10143172

**Published:** 2021-07-19

**Authors:** Magdalena Wujtewicz, Bartosz Regent, Rozalia Marszałek-Ratnicka, Aneta Smugała, Edyta Szurowska, Radosław Owczuk

**Affiliations:** 1 Department of Anaesthesiology and Intensive Therapy, Faculty of Medicine, Medical University of Gdansk, 80-214 Gdansk, Poland; radoslaw.owczuk@gumed.edu.pl; 2 Department of Radiology, University Clinical Centre, The Medical University of Gdansk Hospital, 80-214 Gdansk, Poland; bregent@uck.gda.pl (B.R.); ansmugala@uck.gda.pl (A.S.); 3 Department of Anaesthesiology and Intensive Therapy University Clinical Centre, the Medical University of Gdansk Hospital, 80-214 Gdansk, Poland; rmarszalek@uck.gda.pl; 4 2nd Department of Radiology, Faculty of Health Sciences, Medical University of Gdansk, 80-214 Gdansk, Poland; eszurowska@gumed.edu.pl

**Keywords:** PiCCO, cannulation, artery occlusion, monitoring, ICU

## Abstract

Cardiac output monitoring is a common practice in critically ill patients. The PiCCO (pulse index continuous cardiac output) method requires artery cannulation. According to the manufacturer, the cannula in the radial artery should be removed after three days. However, longer monitoring is sometimes necessary. The aim of this study was to assess the incidence of radial artery occlusion (RAO) after three days of cannulation and to check whether five-day cannulation is related to a higher occlusion rate. An additional assessment was made to verify the presence of occlusion three, fourteen and thirty days after decannulation. The PiCCO cannula was inserted into the radial artery after the Barbeau test and Doppler assessment of blood flow. It was left for three or five days. Doppler was performed immediately after its removal and at three, fourteen and thirty days following decannulation. Thirty-seven patients were randomly assigned for three or five days of cannulation, and twenty-three of them were eligible for further analysis. RAO was found in thirteen (56.5%) patients. No statistical difference was found between the RAO rate for three and five day cannulations (*p* = 0.402). The incidence of RAO was lower when the right radial artery was cannulated (*p* = 0.022; OR 0.129). Radial artery cannulation with a PiCCO catheter poses a risk of RAO. However, the incidence of prolonged cannulation appeared to not increase the risk of artery occlusion. ClinicalTrials.gov ID NCT02695407.

## 1. Introduction

Assessment of the hemodynamics and fluid status of surgical and critically ill patients can be based on different methods and devices. The choice depends on the patient condition, the scope of the required data, or the patient type (e.g., surgical or critically ill) [1,2]. Cardiac output monitoring devices are commonly used in patients treated in the intensive care unit (ICU). The most precise method is direct measurement, which requires artery cannulation. The gold standard is the Swan–Ganz catheter. However, it is a very invasive technique, and its use in the perioperative setting in noncardiac patients is no longer recommended [3]. In critically ill patients, other less invasive techniques have been implemented [4].

PiCCO (pulse index continuous cardiac output) (Pulsion Medical Systems, Germany) is one of the less invasive monitoring techniques, and it is a relatively easy to use tool for determining the main hemodynamic parameters of critically ill patients. It is based on two physical principles, transpulmonary thermodilution and pulse contour analysis. Both principles allow for the calculation of hemodynamic parameters in critically ill patients. The PiCCO method requires cannulation of the central vein and a peripheral artery (i.e., radial, brachial, or femoral). Unfortunately, cannulation may be followed by artery occlusion. In an old literature review, the mean incidence of temporary occlusion of the radial artery when arterial catheters were used for hemodynamic monitoring in anesthesia and intensive care medicine was 19.7% (the incidence ranged from 1.5% to 35%) [5]. According to a prospective observational study by Belda et al., the incidence of radial pulse loss was 3.8% after cannulation with a PiCCO catheter [6].

Generally, data about complications associated with the use of PiCCO arterial catheters are lacking. The most recent information comes from invasive hemodynamic procedures in cardiology. Radial artery occlusion may occur in less than 1% to up to 76% of patients undergoing transradial catheterization for coronary angiography and percutaneous coronary intervention [7,8,9,10]. Arterial cannulation performed in ICU patients lasts much longer than that applied for short hemodynamic diagnostic procedures and, theoretically, the risk of artery occlusion might be higher.

The aim of this study was to verify the occurrence of radial artery occlusion (RAO) after three days of having a PiCCO cannula in place and to check whether five days of cannulation of the radial artery with a PiCCO catheter is related to a higher occlusion rate. An additional assessment was to check whether occlusion was still present three, fourteen and thirty days after decannulation.

## 2. Materials and Methods

This prospective randomized study was approved by the Independent Bioethics Committee for Scientific Research at the Medical University of Gdansk (NKBBN/127/2014). As critically ill patients (due to their condition and sedative drugs) are unable to give an informed consent and the investigated method was a part of patient standard monitoring and forthcoming results of the study could have been helpful in the treatment of critically ill patients, The Committee exempted researchers from the obligation to provide patient informed consent for the study.

Adult patients of both sexes, treated in intensive care unit in of the Medical University of Gdansk hospital, who required vasopressor therapy, were included in the study. Patients were randomly assigned (http://www.randomization.com, accessed on 26 May 2014) to have a cannula in place for either three or five days. The decision to implement the PICCO cannula was based on medical indications.

Preferentially, the nondominant side was chosen and, if not known, it was assumed to be the left side. Barbeau testing and Doppler ultrasonography (Doppler USG) preceded radial artery cannulation. Doppler USG was performed by a radiologist. In cases of Barbeau D status or a lack of flow on ultrasonography, the cannulation was waived.

Cannulation was performed using a dedicated long catheter (4 F, 1.5 mm external diameter, 50 cm long) placed in the radial artery.

Catheter removal (after three or five days of cannulation) was followed by Doppler ultrasonography. Doppler was also performed three, fourteen, and thirty days after decannulation, depending on the patient being available for the assessment.

### Statistical Analysis

The analysis was performed using Statistica (Statsoft, Tulsa, OK, USA) software. The normality of the data distribution was checked with the W Shapiro–Wilk test. Univariate and multivariate logistic regression with a quasi-Newton method of estimation was used to establish factors influencing RAO. Categorical data were analyzed using Fisher Exact test. The level of significance was defined as *p* < 0.05.

## 3. Results

**Figure 1:**
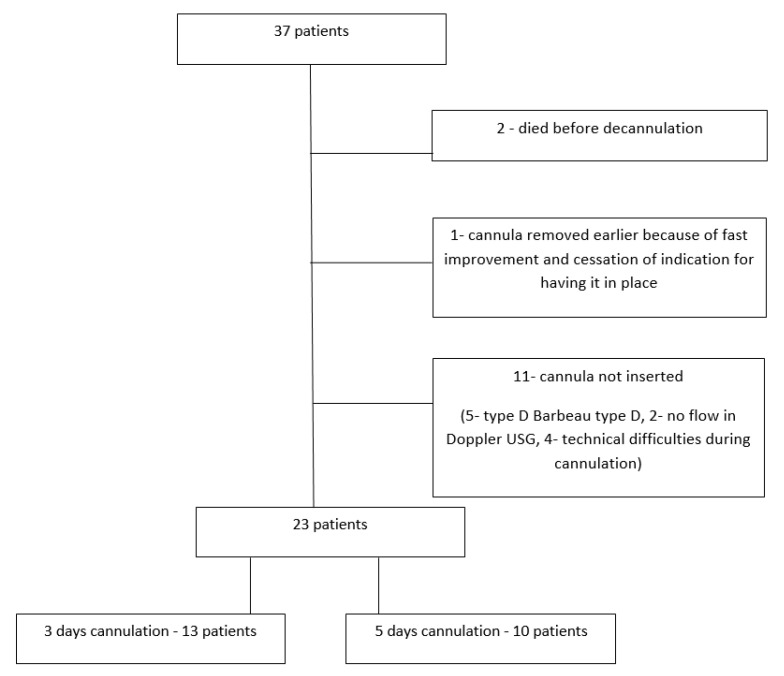
Patients’ flowchart.

Thirty-seven patients were randomly assigned to either three or five days of radial artery cannulation. The patients’ flowchart is shown in Figure 1.

Finally, data from twenty-three patients were analyzed: 56.5% of them had had a cannula in place three days, and 43.5% had a cannula in place for five days. Among these 23 patients, six (26%) were women. The patient demographics are shown in Table 1.

Patient’s basic medical data are shown in Table 2.

Since we use PiCCO monitoring in hemodynamically unstable patients or in those who are stable but require vasopressors, all patients were on vasopressors. One patient in RAO group was on two vasopressors (norepinephrine and epinephrine). The mean dose, described as an equivalent (0.1 μg/kg/min for norepinephrine and epinephrine and 10 μg/kg/min for dopamine) was 1.47 (±SD 1.29) and 1.58 (±SD 1.24) in the group of patients with occlusion after decannulation and in those with present flow, respectively.

After decannulation, RAO was found in 13 (56.5%) patients. In the group with three days of cannulation, the incidence of RAO was 46.1% (6/13 patients). In the group with five days of cannulation, the incidence of RAO was 70% (7/10 patients). No statistical difference was found between the RAO rate for three and five days cannulation (*p* = 0.402)

Three days after decannulation in two cases with previous RAO, blood flow was detected. In four patients, RAO assessment was not possible; three of them died, and one was discharged. Fourteen and thirty days after decannulation of six and two patients, respectively, a previous RAO could be examined and the RAO was still present.

In the group without RAO, three days after decannulation of nine of them, blood flow was present, and one person died before the assessment. Fourteen days after decannulation, three of them were available for assessment and blood flow was present. None of them was available for assessment at thirty days after decannulation.

The comparison of the groups with and without RAO revealed no differences in terms of sex, body mass, height or body mass index. The only factor for which there was a dependence of RAO was the site of cannulation—the incidence of RAO was lower when the right radial artery was cannulated (*p* = 0.022; OR 0.129).

There was no difference in preprocedural Barbeau type, radial artery diameter, blood flow characteristics (flow rate and creases) or vasopressor dose equivalents. One type of the PiCCO catheter was used, with the external diameter 1.5 mm and 500 mm length. The mean radial artery diameter was 2.29 mm (±SD 0.68) and 2.8 (±SD 0.63) in the group with RAO and the group in which the flow was present after decannulation, respectively.

There was no difference in the incidence of RAO in relation to the number of cannulation days. None of the patients had clinical complications of RAO.

## 4. Discussion

ICU and surgical patients often require advanced hemodynamic monitoring, which should be accurate and reliable. For some assessments, such as the patient’s fluid status, less invasive methods can be helpful [11]. However, other patients require more invasive monitoring, for which more precise and invasive tools are necessary. Unfortunately, in Poland, they are not used as often as they should be [12]. According to Lamia et al., five commercially available CO monitoring devices reported similar mean CO values [13]. PiCCO was one of the analyzed devices. It requires cannulation of a ventral vein and an artery. According to the manufacturer, the dedicated cannula in the radial artery should be in place for no longer than three days or in the femoral artery for up to ten days. Unfortunately, in some patients, cannulation of the femoral artery may be difficult or even impossible. Radial cannulation is an alternative, but a three-day period of keeping it in place may not be long enough for some patients.

In our institution, PiCCO is the main hemodynamic assessment tool used in patients treated with vasopressors, and it is implemented in almost all cases. We mostly use femoral access; however, in some patients, such as some trauma victims or morbidly obese patients, radial access is the best choice. Knowledge of the potential incidence of RAO when maintaining radial access for longer than three days might be helpful for making clinical decisions, especially when there is no other way to monitor patient hemodynamics.

In our study, the incidence of occlusion was higher than the incidence of occlusion of the radial artery following transradial interventions in cardiology procedures. This difference may be caused by several factors. First, our patients required invasive monitoring due to hemodynamic instability and the need for vasopressors. Vasopressors cause arterial vasoconstriction. The second reason may be the much longer time, namely days in our study compared to less than 1 h during cardiac diagnostic procedures. In addition, most angiographic cardiology procedures are proceeded by the use of antiplatelet drugs and heparin given during the catheterization. The reason for artery occlusion after cannulation is thrombus formation [14]. It can be caused by direct injury to the vascular wall [15]. None of our patients received a dose of anticoagulation before cannulation like those who undergo cardiac catheterization.

In the study by Garg et al., a radial artery inner diameter ≤2.5 mm and peak systolic velocity were independent predictors of RAO [16]. According to Sinha et al., female sex, diabetes, lower BMI, radial artery diameter ≤2.2 mm, and radial artery-to-sheath ratio (AS ratio) < 1 were predictors of RAO [9]. In our study, only the side of cannulation was linked with a higher risk of RAO. It was the left side. We preferentially chose the nondominant side, and if there were no data about dominance the left side was chosen. However, many patients admitted to our ICU have already had arterial lines placed, so in these cases, we cannulated the other side; 43% of our patients had PiCCO cannulas in the right radial artery.

In terms of safety, there are no differences between left and right radial access for coronary angiography [17]. For diagnostic procedures, it is possible to choose a wider artery of the forearm [18]. Ulnar catheterization is also feasible [19]. The PiCCO cannula is indicated for radial cannulation. Using it for ulnar artery cannulation would be contrary to the manufacturer’s characteristics.

Permanent occlusion of the radial artery after cannulation for monitoring purposes appears to be rare. In a review by Scheer et al., the mean incidence was 0.09%. Temporary occlusion of the artery had no serious sequelae [5]. In our study, RAO was asymptomatic and without any sequelae. This is consistent with results described by others, that most patients with persistent RAO remain asymptomatic. This is due to the dual blood supply to the hand, but this requires a patent ulnar artery [9,16,20]. In our patients, no ischemic events occurred. Small sample size probably influenced on the results and it must be kept in mind, but the lower incidence of RAO in case of right-side cannulation is somehow encouraging, as most of the general human population is right-handed. Eventual adverse, long term consequence of RAO on the left, that would probably result in the worsening of motor function of the hand, could have less influence on patients’ future life/general motor skills.

The lack of ischemic complications despite the high (56%) incidence of RAO in our study may be the result of performing Barbeau testing before cannulation and the withdrawal of cannulation in cases of a D result. The Barbeau test technique has been described elsewhere [21].

In cases of symptomatic RAO, treatment should be started. Therapy with antiplatelet drugs and/or low molecular weight heparin leads to improvement [10]. It is also known that in many cases, spontaneous recanalization of the artery is observed [16]. In our patients, no treatment was used since there were no clinical symptoms such as ischemia. Subjective complaints such as pain or dullness were not possible to verify. In our study, in two cases, the RAO resolved; the lack of blood flow was probably the result of artery constriction. On the other hand, despite the risk of artery occlusion, it is difficult to treat hemodynamically unstable patients without cardiac output or/and systemic vascular resistance monitoring. However, it must be kept in mind that artery cannulation with bore catheters poses some kind of risk. Type of monitoring should be tailored to the patient’s condition and also, as it is especially very important in COVID-19 patients, to the medical staff safety [22].

The most important limitation of the study is a small sample size. The fact, that the only factor for which there was a dependence of RAO was the side of cannulation (lower incidence when right radial artery was cannulated), has no other explanation except the result of small size. It is difficult to find any other explanation. Another limitation of this study is the relatively low number of patients available for follow-up. In the group with occlusion, an assessment fourteen days after decannulation was possible only in six patients, and after thirty days, only two patients were available. In two cases, fourteen days after decannulation the artery re-opened. Two weeks after decannulation six patients were still attainable for evaluation. Thirty days after decannulation there were two patients still hospitalized in the ICU. No new re-opening was noted.

## 5. Conclusions

Radial artery cannulation with a PiCCO catheter poses a risk of RAO. However, the incidence of prolonged cannulation (five days instead of three days) seemed to not increase the risk of artery occlusion. Despite the high incidence of RAO, there were no ischemic complications. The Barbeau test is an easy way of assessing dual blood supply to the hand and should be performed before radial artery cannulation.

## Figures and Tables

**Table 1:** Patient’s demographics.

Parameter	Mean	±SD
Age in years	62.2	18.85
Height in cm	171	7.90
Body mass in kg	78	20.98
BMI in kg m^−2^	26.5	6.25
Initial diameter of the artery in mm	2.51	0.72

BMI, body mass index.

**Table 2:** Patient’s groups medical data.

Parameter	Group with RAO (*n* = 13)	Group without RAO (*n* = 10)
Vasopressors use	yes (13/13)	yes (10/10)
LMWH use	yes (12/13)	yes (9/10)
-patients with therapeutic LMWH dose	0/13	1/10
Antiplatelet drug use	no	no
Anticoagulation drugs	no	no
Basic diagnosis		
-TBI	3/13	0/13
-polytrauma	1/13	1/10
-respiratory insufficiency (pneumonia)	3/13	3/10
-respiratory insufficiency (other than pneumonia)	0/13	2/10
-cardiac arrest	4/13	2/10
-septic shock	2/13	1/10
-hemorrhagic shock	0/13	1/10

LMWH, low molecular weight heparin; TBI, traumatic brain injury.

## Data Availability

All the data supporting the findings of this study are available within the article.
